# Advancing Albumin Isolation from Human Serum with Graphene Oxide and Derivatives: A Novel Approach for Clinical Applications

**DOI:** 10.1021/acsomega.4c04276

**Published:** 2024-09-20

**Authors:** Chayachon Apiwat, Jack W. Houghton, Ren Ren, Edward Tate, Joshua B. Edel, Narong Chanlek, Patraporn Luksirikul, Deanpen Japrung

**Affiliations:** † Department of Chemistry, Faculty of Science, Kasetsart University, Bangkok 10900, Thailand; ‡ Center for Advanced Studies in Nanotechnology for Chemical, Food and Agricultural Industries, KU Institute for Advanced Studies, Kasetsart University, Bangkok 10900, Thailand; § National Nanotechnology Center (NANOTEC), National Science and Technology Development Agency (NSTDA), Thailand Science Park, Pathumthani 10120, Thailand; ∥ Synchrotron Light Research Institute (Public Organization), 111 University Avenue, Muang, Nakhon Ratchasrima 30000, Thailand; ⊥ Department of Chemistry, Molecular Sciences Research Hub, Imperial College London, London W12 0BZ, U.K.; # Department of Metabolism, Digestion and Reproduction, Imperial College London, London SW7 2AZ, U.K.

## Abstract

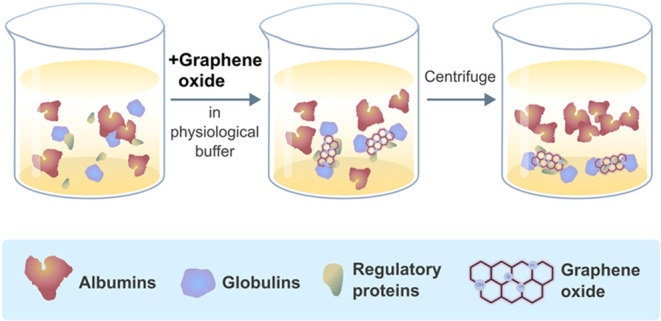

This study introduces a novel, environmentally friendly albumin isolation method using graphene oxide (GO). GO selectively extracts albumin from serum samples, leveraging the unique interactions between GO’s oxygen-containing functional groups and serum proteins. This method achieves high purification efficiency without the need for hazardous chemicals. Comprehensive characterization of GO and reduced graphene oxide (rGO) through techniques such as X-ray diffraction (XRD) analysis, Raman spectroscopy, scanning electron microscopy (SEM), and Fourier transform infrared spectroscopy (FTIR) confirmed the structural and functional group transformations crucial for protein binding. Sodium dodecyl sulfate–polyacrylamide gel electrophoresis (SDS-PAGE) and mass spectrometry analyses demonstrated over 95% purity of isolated albumin, with minimal contamination from other serum proteins. The developed method, optimized for pH and incubation conditions, showcases a green, cost-effective, and simple alternative for albumin purification, promising broad applicability in biomedical research and clinical applications.

## Introduction

Albumin, a single-chain, water-soluble protein encompassing over 500 amino acids with a molecular weight of approximately 67 kDa, is found in blood plasma or serum. Its significant applications across pharmaceutical and medical science technologies highlight its versatility, serving as a drug delivery system, in infusion therapy, as an antioxidant, a corrosion inhibitor, and as a blocking and supporting material for biosensors to identify or detect analytical compounds. Recent studies have demonstrated the potential of albumin in novel drug delivery systems and regenerative medicine, highlighting its expanding role in biomedicine.^1−3^ Albumin sources are diverse, including human and animal origins, leading to various types such as human serum albumin (HSA) and bovine serum albumin (BSA), depending on their species’ origin. While plants do not naturally produce albumin, genetically modified plants, such as rice, have been engineered to produce recombinant albumin. Specifically, HSA, extracted from human plasma, maintains a concentration of 0.6 mM or 30–50 g/L in healthy adults.^4^ Changes in albumin levels in the human body can indicate various conditions and diseases, including acute liver failure, shock, burns, hypovolemia, and hypoproteinemia.^5−7^ HSA plays a crucial role in maintaining oncotic blood pressure and pH levels, and it primarily binds and transports low molecular weight molecules of various origins. Albumin itself plays a crucial role in these regulatory functions. Additionally, it aids in antioxidant activity, affects capillary membrane permeability, and offers a neuroprotective effect, highlighting its varied roles in physiological mechanisms. Due to albumin’s significant physiological and biopharmaceutical functions, numerous efforts have been made to produce high-purity and high-quality albumin. There is an increasing demand for its use in clinical practices and research methodologies.^8−15^ Purified albumin can be obtained through various methods, including chromatography, solvent extraction, and adsorption.^16^ Currently, conventional albumin purification methods utilize a combination of the Cohn method and various chromatography techniques. The Cohn method, developed by Edwin J. Cohn, is a fractionation process that uses ethanol, pH, temperature, and ionic strength to precipitate and separate plasma proteins, including albumin, into different fractions.^17^ These methods are valued for producing high-purity and quality products. However, they are complex, costly, time-consuming, and require hazardous chemicals. Furthermore, their operation is intricate and demands specialized skills.^5,17−27^ Therefore, this study explores an alternative, novel, and straightforward method for albumin purification.

Graphene, graphene oxide (GO), and reduced graphene oxide (rGO) are two-dimensional carbon nanomaterials that have attracted significant scientific interest due to their unique physicochemical properties. These properties include a high aspect ratio, ultrahigh strength, exceptional thermal conductivity, and electrical conductivity, in the case of rGO.^28−30^ They are promising materials for a wide range of applications, such as in electronics, energy storage and conversion, catalysis, and sensors, including fields related to protein binding or purification.^31−33^ GO contains a graphene basal plane with oxygenated functional moieties, such as carboxyl, hydroxyl, epoxy, and other functional groups. It can be prepared through the oxidation of graphite with strong acids, resulting in an oxidized graphene sheet with expanded interlayer distances between graphitic sheets. These structural changes render GO hydrophilic, allowing it to form stable aqueous colloids that facilitate simple biological solution processes or become biocompatible. The oxygen functional groups on the graphene sheet can also provide various active reaction sites for attaching analytical species. In addition, rGO, a chemically derived graphene, can be directly obtained from the chemical or thermal reduction of GO. The chemical or thermal treatment of GO reduces it to graphene-like sheets by removing oxygen-containing groups, recovering a conjugated graphene sheet structure. The structure of rGO varies, altering the residual functional groups and defects that affect its chemical properties. Consequently, the properties of GO and rGO are substantially different.

In recent studies within medical and biological fields, GO has gained significant interest due to its interaction with proteins, particularly those found in biological fluids such as blood plasma or serum. These fluids comprise albumin, globulins, fibrinogen, and various regulatory proteins.^34−40^ Upon encountering biological fluids, GO attracts proteins to its surface, forming what is known as a “biomolecular corona” or “protein corona”.^41^ The composition of this corona varies based on factors like particle type, surface charge, pH, and size distribution.^36−39,41^ Interestingly, studies on protein coronas have shown a tendency for globulins to bind to GO surfaces more than albumin under certain conditions.^42−47^

Herein, we introduce an environmentally friendly, one-step albumin purification method that avoids using hazardous chemicals. This process employs GO sheets in neutral phosphate-buffered saline to extract albumin from serum, taking advantage of the negative charge of oxygen-containing functional groups on the graphene derivative. The hexagonal aromatic graphene structure also facilitates various interactions with serum proteins through hydrogen bonding, electrostatic forces, hydrophobic effects, and π–π interactions. GO was specifically chosen for a systematic study to identify the optimal conditions for albumin purification. Varying the pH values of GO solutions alters protein-binding properties, aiding in the selective removal of unwanted serum proteins. By optimizing incubation time and sample-to-GO solution ratios, we were able to isolate albumin with high purity, confirmed via mass spectrometry (MS) analysis.

## Materials and Methods

### Materials and Chemicals

Graphite flakes (99% carbon basis, ∼325 mesh), hydrazine monohydrate (N_2_H_4_, 64–65%), and potassium bromide (KBr, ≥99%) were purchased from Sigma-Aldrich (St. Louis). Potassium permanganate (KMnO_4_) was purchased from KEMAUS (NSW, Australia). Sulfuric acid (H_2_SO_4_, 95–97%), phosphoric acid (H_3_PO_4_, 85%), hydrochloric acid (HCl, 37%), and hydrogen peroxide (H_2_O_2_, 30%) were purchased from Merck (Germany). Deionized (DI) water was used both for cleaning during synthesis and for preparing reagent solutions. HSA and lysozyme were purchased from Sigma-Aldrich Co. (St. Louis, MO). A solution of 40% Acrylamide/Bis (29:1) and Precision Plus Protein Standards was sourced from Bio-Rad Laboratories, Inc. (Hercules, CA). All chemicals used in this study are analytical grade.

### Synthesis of Graphene Oxide

The GO used in this study was synthesized using an improved Hummers method.^48^ Briefly, a mixed solution of sulfuric acid (H_2_SO_4_) and phosphoric acid (H_3_PO_4_) (200 mL) was prepared at a volume ratio of 9:1. Graphite flakes (1.5 g) were then gradually added to this solution under stirring, followed by the slow addition of potassium permanganate (9.0 g). The mixture was subsequently heated to 50 °C and stirred for 12 h. Upon completion of the reaction, the mixture was poured onto ice (∼200 mL) with 30% H_2_O_2_ (3 mL) and allowed to cool to room temperature (RT). The resulting solid was washed repeatedly with 5% HCl to remove residual metal oxides and then with deionized water until the runoff was neutral. After each washing step, the mixture was centrifuged at 7000 rpm for 10 min. Finally, the solid was vacuum freeze-dried overnight to yield the GO particles.

### Synthesis of Reduced Graphene Oxide from Graphene Oxide

To chemically reduce the oxygen content in GO, a procedure outlined in references^49,50^ was followed. Initially, 50 mg of synthesized GO was dispersed in 9 mL of deionized water. This mixture underwent sonication in an ultrasonic bath to ensure uniform dispersion. Subsequently, 1 mL of 10% v/v hydrazine monohydrate was introduced to the solution. The mixture was then subjected to reflux at 100 °C for 1 h in an oil bath to reduce the oxygen functional groups present in the GO. As a result, the solution darkened, signifying the formation of a black solid indicative of the reduction process. This solid was isolated using centrifugation at 6000 rpm for 10 min and was purified through repeated washing with deionized water, ensuring the removal of unreacted materials. Each washing cycle involved centrifugation under the same conditions. The resultant rGO was then dried at 70 °C overnight, yielding a fine powder ready for further analysis.

### Characterization Techniques for Graphene Oxide and Reduced Graphene Oxide Particles

Scanning Electron Microscopy (SEM) FEI Quanta 450 and Transmission Electron Microscopy (TEM) JEOL 2100 were employed to examine the microstructure and surface morphology of GO and rGO particles. Energy dispersive X-ray spectroscopy (EDS or EDX) mapping was performed inside the SEM for quantitative elemental analysis in the as-prepared samples. A Bruker D8 Advance powder diffractometer for X-ray (XRD) diffractograms of graphene-based materials and charcoal was used, employing Cu Kα radiation and scanning in the 2theta range from 5 to 50°. Infrared spectra were measured using a Bruker Tensor 27 Spectrometer, with KBr as a reference, across a 500–4000 cm^–1^ scan range. A HORIBA LabRAM HR Evolution Confocal Raman Microscope was utilized to measure Raman spectra, determining the microstructures and the presence of functional groups on graphene and all samples. Thermal Gravimetric Analysis (TGA) measurements were conducted using a PerkinElmer Pyris 1 thermal analyzer at a 5 °C/min heating rate under airflow. The Malvern Nano ZS Zetasizer was used for ζ-potential and hydrodynamic size distribution data of protein-bound and naked materials. X-ray photoelectron spectroscopy (XPS) spectra of graphene derivatives were recorded using a PHI5000 VersaProbe II (ULVAC-PHI, Japan) at the SUT-NANOTEC-SLRI Joint Research Facility, Synchrotron Light Research Institute (SLRI), Thailand. A monochromatized Al–Kα X-ray source (1.486.6 eV) was used as an excitation source. The XPS spectra were fitted with a combination of Gaussian–Lorentzian curves to determine the chemical compositions and C/O atomic ratio of graphene derivatives.

### Optimization of pH for Protein Binding Studies Using Graphene Oxide and Reduced Graphene Oxide

The buffer solutions were prepared separately: sodium citrate buffer with pH ranging from 4.5 to 5.5, and phosphate-buffered saline (PBS) with pH ranging from 6.0 to 7.0. These buffers were used to optimize the pH for protein binding studies using GO and rGO. A solution of 0.6 N HCl is prepared to adjust the pH of the PBS solution. The separate buffers to cover all used pH range were prepared. The most suitable material and solution are selected by comparing the protein-binding abilities of the graphene derivative materials. Briefly, GO and rGO solutions, at pH 7.0, were preliminarily tested with known net-charged proteins. SDS-PAGE analysis and DLS studies were conducted for measurements. The pH of the solution is determined by preparing a series of GO solutions with varying pH values ranging from 4.5 to 7.0.

### Development of Albumin Extraction Method Using Graphene Oxide

An albumin extraction method was developed using GO as the extractant reagent. The extraction parameters such as pH value, initial concentration of the extractant (the volume ratio between extractant (GO) and serum sample), and extraction time were carefully optimized. Specifically, a stock GO aqueous solution (2 mg/mL, pH 6.5) was employed as the extractant solution. Different volumes, ranging from 20 to 100 μL, were mixed with a constant serum sample (EC approval number: 431/2557) volume (2 μL) to identify the optimal volume ratio of extractant reagent to serum sample. Following this, the mixture was incubated for 15 min at room temperature. To fine-tune the extraction time, incubation periods were varied from 15 min to 24 h.

For comparison, the serum samples were processed using the charcoal-based extraction step from the EXOCELL Glycaben assay. This step is designed to remove hydrophobic molecules bound to albumin but does not significantly purify albumin from other proteins. Specifically, 2 μL of the serum sample was mixed with 20 μL of the charcoal-based extraction buffer and incubated overnight at room temperature. The mixture was then centrifuged at 14,000 rpm for 10 min, and the supernatant was collected for SDS-PAGE analysis. The limitations of this method in achieving high-purity albumin are acknowledged and discussed in the Results and Discussion section.

**Figure 1:**
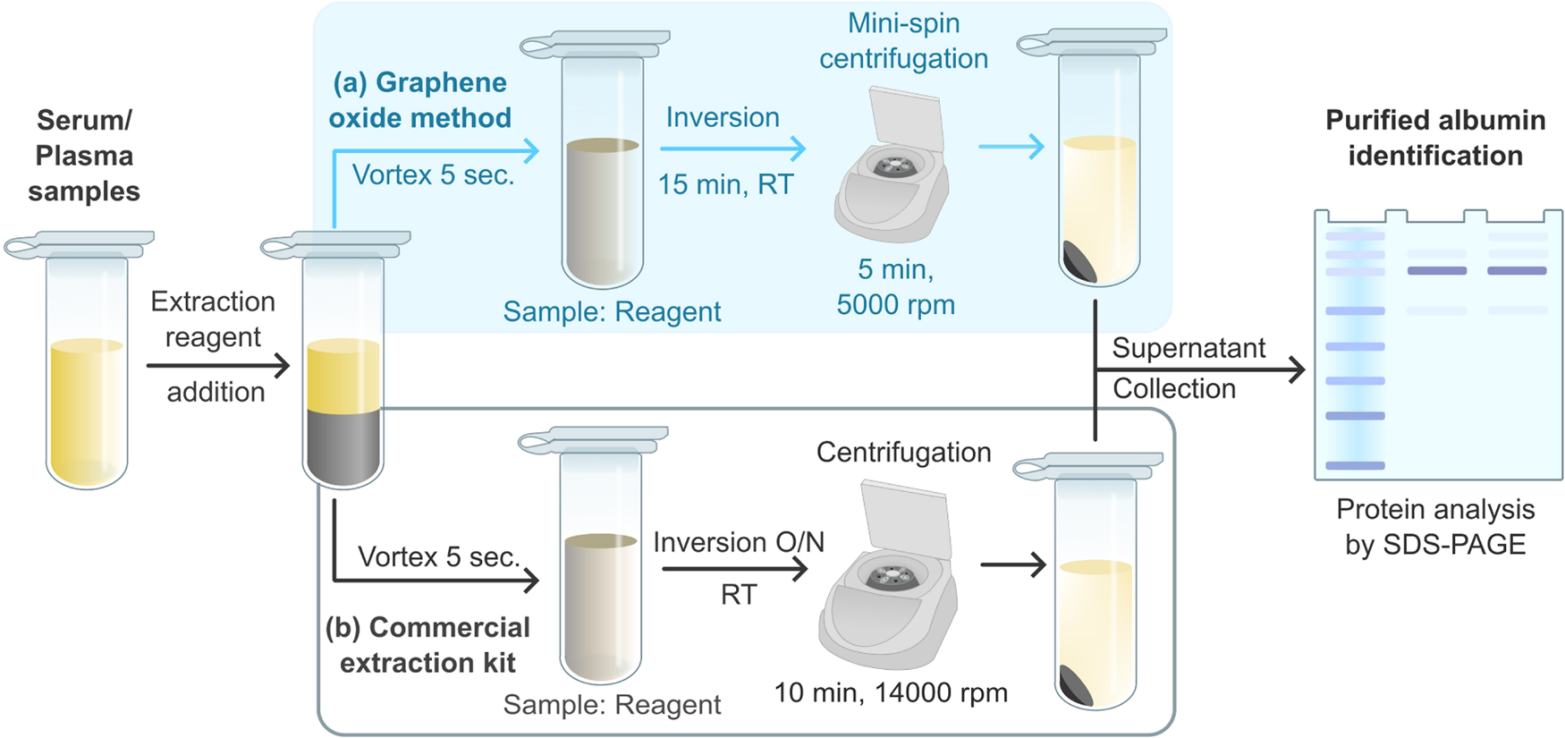
Comparative schematic of albumin isolation protocols. (a) Developed GO-based method entails the addition of GO extraction reagent to serum/plasma, followed by a 5 s vortex, a 15 min room temperature incubation, and a 5 min centrifugation at 5000 rpm. The supernatant is then analyzed for albumin purity via SDS-PAGE. (b) The commercial kit method begins similarly with reagent addition and vortexing, followed by 10 min centrifugation at 14000 rpm, with the final albumin purity also assessed by SDS-PAGE.

The purity of the extracted albumin in the supernatant was evaluated using SDS-PAGE analysis. The efficiency of this method was compared to that of a charcoal-based extraction buffer from the EXOCELL Glycaben assay, a commercially available kit. A schematic diagram illustrating the albumin extraction process is presented in Figure 1.

### Analysis of Albumin Protein Using SDS-PAGE

The purity of the albumin fraction was examined using sodium dodecyl sulfate–polyacrylamide gel electrophoresis (SDS-PAGE) under reducing conditions. A 12% SDS-PAGE gel was run at a constant voltage (100 V) for 1.40 h, followed by staining with Coomassie Brilliant Blue R-250 (0.25% w/v in 45% methanol, 10% acetic acid) for 30 min for protein band visualization.^9,51^ The gels were then destained three times in a solution of 40% methanol and 10% acetic acid overnight until the protein bands were observed. Each lane was loaded with 2 μg of total protein. The albumin band densities were analyzed using ImageJ for relative quantification of protein bands. The “+” or “–” symbols in the results represent the presence or absence of proteins rather than precise concentration measurements.

### Proteomic Analysis of Serum Albumin Purity and GO Method Efficiency

Label-free mass spectrometry-based quantitative proteomics was conducted to examine the purity of purified serum albumin and evaluate the efficiency of the GO extraction method. Proteins in the GO-purified serum samples were prepared for peptide analysis as follows: after completing the GO method, the supernatants were collected and subjected to an albumin depletion step using the Pierce Albumin Depletion Kit 85160 (Thermo Scientific). For proteomic sample preparation, the as-prepared supernatants were transferred to fresh low-bind microcentrifuge tubes. Then, 0.5 volumes of 40 mM TCEP were added, followed by 0.5 volumes of 160 mM CAA; both buffers were prepared in 25 mM AMBIC (Ammonium bicarbonate), which has a pH of approximately 7.8–8.0, the optimal pH for trypsin activity. The mixtures were shaken at room temperature (RT) for 10 min on an Eppendorf shaker. Following the SP4 (Solvent Precipitation 4) protocol, ACN was added to the mixture at a volume ratio of 4:1 and gently vortexed for 5 s. SP4 employs the capture of acetonitrile-induced protein aggregates through a centrifugation process using glass beads or bead-free. This technique is acknowledged for its ability to isolate low-solubility aggregates that often include insoluble transmembrane proteins.^52^ Protein pellets were collected by centrifugation at 14,680 g for 5 min, followed by three washes with 80% ethanol. More than 95% of the supernatant was removed for the final wash, the remaining pellet was then prepared for digestion. Trypsin, in 25 mM AMBIC at a final concentration of 6.25 ng/μL, was prepared as a digestion buffer and added (25 μL) to each pellet sample. Proteins were digested on a thermomixer at 600 rpm for 18 h at 37 °C in the dark. The peptide mixture was then collected, quantified by NanoDrop, dried in a speed vacuum, and reconstituted in 20 μL of rehydration solution (2% ACN and 0.5% TFA in LC-MS grade water).

LC-ESI-MS/MS analysis was performed on a Q-Exactive Plus mass spectrometer coupled with an Easy-nLC 1000 HPLC system (Thermo Fisher Scientific). Briefly, 3 μL (equivalent to 0.4 μg) of the peptide mixture was first injected into a trap column (100-μm internal dimension × 2 cm, Acclaim PepMap 100 Precolumn, Thermo Fisher Scientific) in solvent A (0.1% formic acid in water). Reversed-phase high-performance LC was then carried out using the Easy-nLC 1000 HPLC system with an analytical column (75-μm internal dimension × 50 cm, 2-μm particle, Acclaim PepMap RSLC C18, Thermo Fisher Scientific). The peptides were separated by a 70 min analytical gradient from 5% to 35% solvent B over 45 min, rising to 99% solvent B by 5 min, followed by an 18.5 min wash at 99% solvent B. The columns were maintained at 40 °C. MS/MS data acquisition was in data-dependent mode with specified settings: MS1 window of 375–1650 *m*/*z*, resolution of 70,000, AGC target of 1 × 10^6^, and maximum injection time of 20 ms; MS2 settings included quadrupole isolation with a width of *m*/*z* 2, high-energy collisional dissociation (NCE 25), fragment ions scanning from *m*/*z* 120 in the Orbitrap, AGC target of 1 × 10^5^, and maximum injection time of 120 ms. Dynamic exclusion was set to ±10 ppm for 20 s, and MS2 fragmentation was triggered on precursors with counts of 1 × 10^2^ and above.

Raw MS data were processed using MaxQuant v2.2.0.0 and searched against the UniProtKB *Homo sapiens* proteome (downloaded on 1/12/2022 from http://www.uniprot.org). Label-free quantification was performed with standard settings; the main search peptide tolerance was set to 4.5 ppm. Trypsin/P was specified as the digesting enzyme, with up to two missed cleavages allowed. Oxidation of methionine and protein N-terminal acetylation were treated as variable modifications, and carbamidomethylation of cysteine residues was a fixed modification. A maximum of five modifications per peptide was permitted. Only peptides with a minimum of seven amino acids and at least one unique peptide were considered for protein identification. Intensity-based absolute quantification (iBAQ) values, calculated from ion intensities, were used to estimate the relative abundance of proteins in each sample. A pie chart illustrates the percentage of each protein remaining in the purified sample.

## Results and Discussion

### Structural Analysis and Characterization of Synthesized Graphene Oxide and Reduced Graphene Oxide

**Figure 2:**
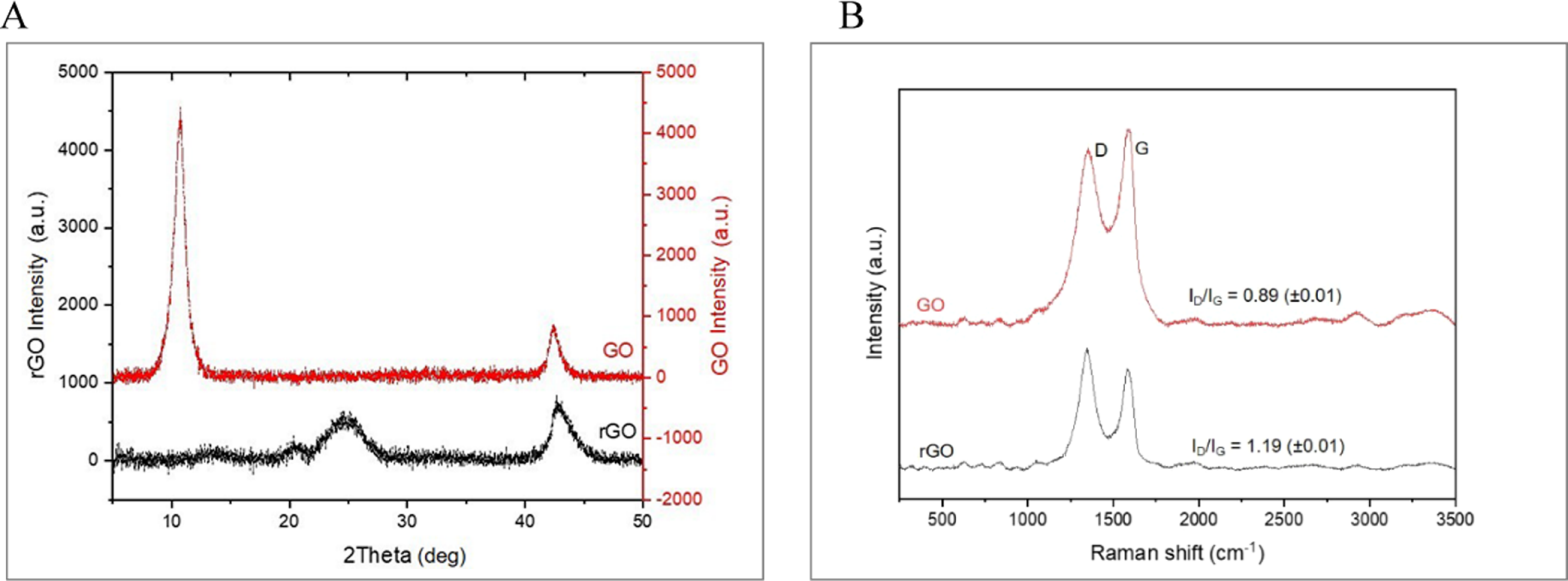
Comparative analysis of GO and rGO. (A) shows the XRD patterns of as-prepared GO (red line) and rGO (black line), illustrating distinct peak shifts that indicate differences in their crystal structures. (B) presents the Raman spectra for GO (red line) and rGO (black line), highlighting the characteristic D and G bands. The *I*_D_/*I*_G_ ratios, indicative of disorder within the graphitic structure, are annotated for both materials.

GO and rGO were successfully synthesized as described in the Materials and Methods section. The XRD spectra of the as-synthesized GO and rGO are displayed in Figure 2A, and the structural parameters obtained from the XRD results are shown in Table 1. Theoretically, the XRD pattern of graphite exhibits a characteristic sharp peak (002) with high diffraction at a 2θ of 26.62°, and an interlayer distance (*d*-spacing) of 0.33 nm.^53,54^ Due to the formation of oxygenated groups on the graphite surface from oxidizing agents during the oxidation reaction, the peak (002) shifts to 2θ = 10.68° in the GO sample, confirming an increase of *d*-spacing to 0.8 nm indicative of the intercalation of oxygen functional groups such as hydroxyl, epoxide, and carboxylic groups.^55,56^ The rGO (002) reflection (2θ = 25.12°, *d*-spacing = 0.4 nm) is close to that of graphite, indicating that hydrazine monohydrate acts as an effective reductant. The changes in GO structure due to chemical treatment are also confirmed by Raman spectroscopy analysis.

**Table 1:** Comparative Structural Parameters of GO and rGO Derived from XRD Analysis^a^

	**peak (002)**					**peak (100)**		
**sample**	**2θ [deg]**	**fwhm [deg]**	***H* [nm]**	***d*****[nm]**	***n***	**2θ [deg]**	**fwhm [deg]**	***D* [nm]**
GO	10.68	1.10	7.88	0.827	9–10	42.40	1.09	8.5
rGO	25.12	3.14	2.82	0.354	8	42.77	1.74	5.3

a The angle of the diffraction peak, represented as 2θ [deg], indicates the crystal orientation. The peak width at half its maximum height denoted as fwhm [deg], is related to the size of the crystallites. The height of the (002) peak, labeled *H* [nm], can suggest the stack’s thickness. The distance between the crystal layers, shown as *d* [nm], reveals the spacing within the crystal structure. The parameter “*n*” estimates the number of layers in the material. Finally, *D* [nm] refers to the size of the coherent crystalline domains.

The Raman spectroscopy method is a prominent tool for characterizing defects, changes in the graphene layer structure, and the crystalline size of graphitic materials.^57^Figure 2B shows the Raman spectra of GO and rGO, which exhibit two characteristic peaks of graphene-based materials, positioned at around 1350 cm^–1^ (D band) and 1580 cm^–1^ (G band), characteristic of graphene-based materials.^58,59^ The G band is related to the presence of an sp^2^ carbon network, while the D band is attributed to structural defects or oxidation in the graphitic materials.^53,60,61^ The appearance of the D band in the as-prepared GO sample indicates that the introduced oxygen functional groups disrupt the original lattice structure of the graphite precursor.^62,63^ Typically, the disorder in these graphitic materials can be estimated by the ratio *I*_D_/*I*_G_.^53,60^ The precursor material, graphite flakes used in this work, has a ratio *I*_D_/*I*_G_ of 0.20 ± 0.07, which suggests that the structures of GO and rGO are more disordered and possess more defects through the layers compared to graphite. Furthermore, the defects or disorder on these layers increase after treatment with reductants. The increase in disorder after the reduction process might be correlated with the removal of internal moieties in the graphene net, leading to defects or holes,^60^ making rGO more disordered than GO, as detailed in Table 2.

**Table 2:** Raman Spectroscopic Analysis of GO and rGO^a^

	**peak position**		**peak intensity**		
**sample**	**D-band [cm^–1^]**	**G-band [cm^–1^]**	**D-band [au]**	**G-band [au]**	***I***_**D**_**/*I***_**G**_
GO	1356	1587	1493.32	1677.24	0.89 (±0.01)
rGO	1346	1580	1036.38	863.56	1.19 (±0.01)

a The D-band (∼1350 cm^–1^) and G-band (∼1580 cm^–1^) reflect graphene’s structural aspects; the D-band signals disorder, and the G-band indicates sp^2^ carbon. Intensity in arbitrary units (au) shows feature strength. The *I*_D_/*I*_G_ ratio, assessing disorder, reveals more defects with higher values, contrasting GO and rGO’s structural changes postreduction.

### Microscopic Analysis of Graphene Oxide and Reduced Graphene Oxide Structures

**Figure 3:**
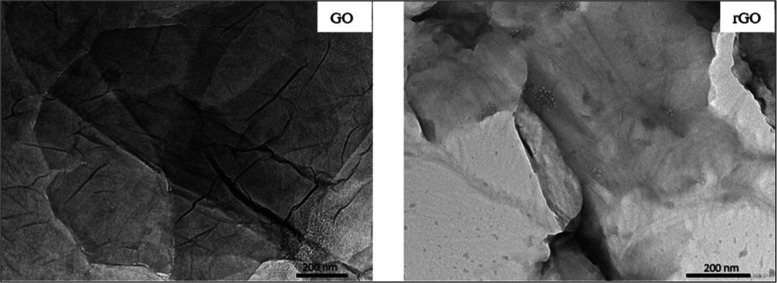
TEM images showcase the morphological differences between GO on the left, characterized by its transparent and wrinkled surface, and rGO on the right, which displays a more opaque and densely folded texture. The contrast highlights the impact of reduction on GO’s structural properties.

TEM and SEM were utilized to examine the structural quality and morphology of as-prepared GO and rGO, respectively. TEM images, presented in Figure 3, are formed as electrons interact with the material during transmission. GO’s TEM image reveals greater transparency and a more wrinkled surface, indicating varying thickness and the presence of oxygen functional groups, or the oxidation level in the GO sample. In contrast, rGO appears with opaque, dense flakes, exhibiting rough surface folds akin to an amorphous structure, with fewer wrinkles and less transparency.

**Figure 4:**
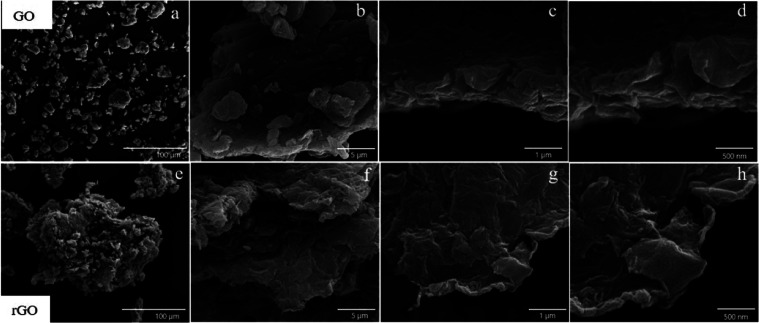
Comparative SEM analysis of GO and rGO. Panels (a–d) showcase GO at varying magnifications, highlighting its layered and wrinkled surface structure. Panels (e–h) display rGO, revealing surface texture and morphology changes due to reduction, including increased folding and amorphization.

Figures 4 and S1 display the SEM images of GO and rGO composites. GO’s surface features a wrinkled edge and is relatively smooth, highlighting a blend of carbon and oxygen within a layered structure. The rGO images showcase that the removal of most oxygen-containing functional groups during the reduction process results in a more wrinkled, folded texture, and amorphization. Although the SEM images present the surface morphologies of GO and rGO as not markedly different, the presence of abundant oxygen functional groups in the GO structure has been further verified by FTIR, XPS, EDX, and TGA analyses. The values reported in Figure S1 were determined in this work. These values (elemental composition) were obtained using the SEM-EDX (scanning electron microscopy with energy dispersive X-ray spectroscopy) technique. SEM-EDX provides surface composition information on the surveyed area, and EDX analysis coupled with SEM was used to determine the atomic mass of the elements in the samples. The lower the peak response, the lower the atomic mass. The abundance of elements in the sample corresponds to the size of the detecting peak, which displayed X-ray spectra generated from the entire scanned areas of SEM images (Figure S1).

### Spectroscopic Analysis of Functional Group Transformations in Graphene Oxide and Reduced Graphene Oxide

**Figure 5:**
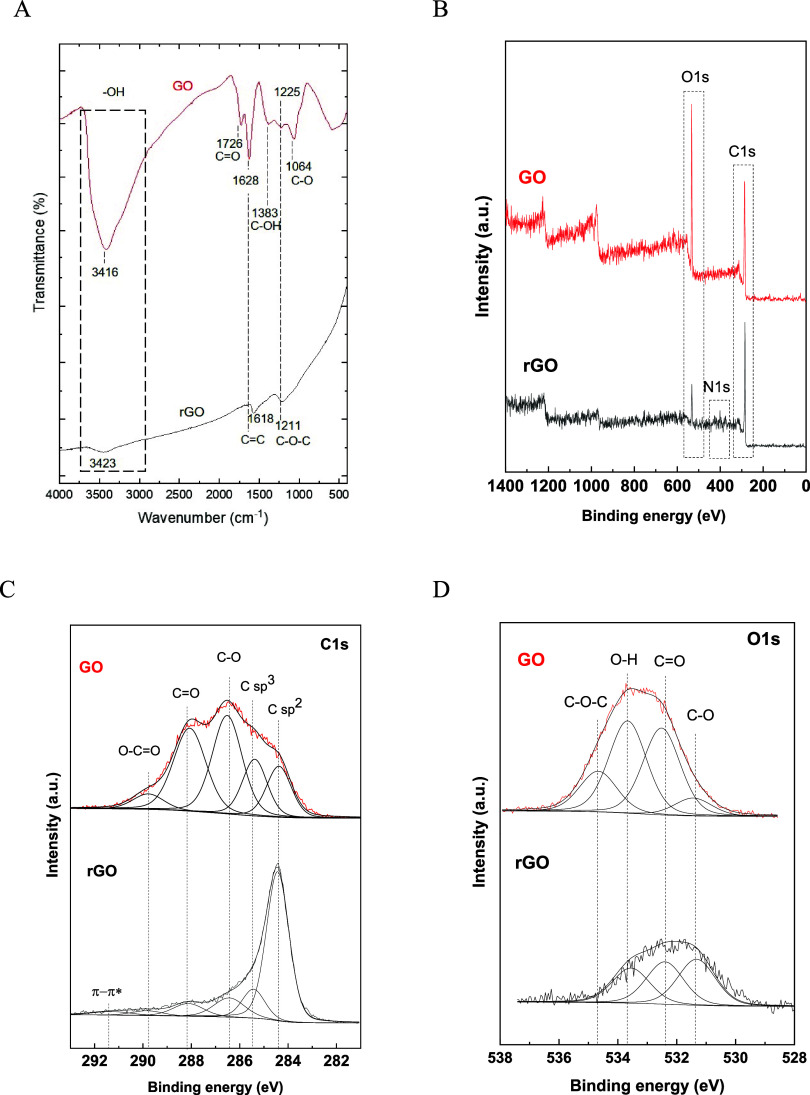
Comparative analysis of GO and rGO functional groups. (A) FTIR spectra highlight characteristic functional groups, with notable differences in −OH, C=O, and C–O stretching vibrations between GO and rGO. (B) Wide scan XPS spectra provide an overview of elemental composition, showing distinct peaks for C 1s and O 1s. (C) Deconvoluted C 1s XPS spectra of GO and rGO detail the presence and reduction of carbon-associated functional groups. (D) Deconvoluted O 1s XPS spectra reveal changes in oxygen-containing groups, indicating an effective reduction in rGO.

The presence of various oxygen functional groups in GO and rGO was analyzed using FTIR spectroscopy. FTIR spectra, shown in Figure 5A, revealed a typical transmission band around 1620 cm^–1^ in both GO and rGO spectra, attributed to physisorbed water through hydrogen bonding.^64^ A broad peak at approximately 3400 cm^–1^ in GO, and a flattened peak in rGO, indicate the presence of −OH groups. Postreduction, the flattened peak in rGO suggests that not all hydroxyl groups are eliminated, supported by the hydroxyl group bending vibration around 1220 cm^–1^. FTIR analysis also identified other characteristic peaks for GO, including C=O stretching at approximately 1720 cm^–1^, C–O stretching at around 1049 cm^–1^,^65^ and a carboxyl group at 1383 cm^–1^, indicating that epoxy and phenolic hydroxyl groups are located in the basal plane, while carboxyl groups are at the edges of GO sheets, formed during graphite oxidation.^64,66^ The rGO spectrum showed a reduction in these oxygenated groups, including the disappearance of the carbonyl group at approximately 1738 cm^–1^, carboxyl group at 1383 cm^–1^, and epoxy group at around 1064 cm^–1^,^64^ confirming the reduction of GO to rGO with hydrazine monohydrate.

XPS analysis further examined the chemical composition and state of the samples. The XPS spectra in Figure 5B for GO and rGO revealed intense peaks corresponding to C 1s and O 1s, with the elemental composition shown in Table 3. The oxygen content in rGO significantly decreased compared to GO, demonstrating the reduction process’s effect. Additionally, in the hydrazine-reduced sample, nitrogen constitutes 3.47% of the total atomic composition (%), signifying the incorporation of nitrogen atoms into the material’s structure during the reduction process. The deconvoluted C 1s XPS spectra for GO, illustrated in Figure 5C, comprise five peaks corresponding to various carbon bonds and functional groups.^67−70^ The details of atomic percentage area (at.%area) are shown in Table S1. It showed the peaks located at binding energies of ∼284.4, 285.7, 286.6, 287.9, and 289.6 eV, which correspond to the carbon sp^2^ hybridization (nonoxygenated C, C=C), C sp^3^, C–O, C=O, and O–C=O groups, respectively of the GO. On the other hand, the deconvoluted C 1s peaks with binding energies of 284.4, 285.5, 286.6, 288.0, 289.6, and 291.3 eV, which are attributed to the carbon sp^2^ hybridization, C sp^3^, C–O, C=O, O–C=O groups, and π–π* satellite bonds, respectively for the rGO sample.

**Table 3:** Elemental Composition and C/O Atomic Ratios in GO and rGO by XPS and EDX-SEM

element	GO XPS (%)	GO EDX-SEM (%)	rGO XPS (%)	rGO EDX-SEM (%)
C	66.14	64.76	84.91	88.49
O	33.86	35.24	11.62	11.51
N	-	-	3.47	-
C/O ratio	1.95	1.84	7.31	7.69

Figure 5D also show a high resolution XPS spectra of O 1s. The deconvoluted O 1s peaks with binding energies of 531.5, 532.5, 533.7, 534.7 eV, which represent to C–O, C=O, C–OH, C–O–C groups, respectively for GO. However, the deconvoluted of O 1s phase for rGO have the binding energies of 531.3, 532.4, 533.6 eV, assigned to the C–O, C=O, C–OH groups, respectively. These suggest that the most oxygenated functional groups containing in the as-prepared GO are carboxylic (C=O and O–C=O) and hydroxyl (−OH) groups. After reduction, the rGO C 1s spectrum showed a reduction in peak intensities of oxygen-containing functional groups due to the removal of oxygenated groups by hydrazine monohydrate. Interestingly, some literature^71−73^ suggests hydrazine hydrate cannot reduce carboxylate and carbonyl groups, yet Figure 5D also indicates a significant reduction, suggesting these groups can be reduced by hydrazine. This finding aligns with an open question on whether hydrazine can remove C–OH groups from GO.^68^ The chemical states identified via XPS closely correlate with the FTIR results.

The unusual XPS spectrum for the C 1s region in our GO sample may be due to the presence of higher oxidized structure of GO resulting in broadening the C–C/C=C peak. After acid treatment by improved Hummer method, graphite was completely oxidized given as GO sample. Due to the formation of oxygenated groups on the graphite surface from oxidizing agents during the oxidation reaction, the peak (002) shifts to 2θ = 10.68° in the GO sample, confirming an increase of *d*-spacing to 0.8 nm indicative of the intercalation of oxygen functional groups such as hydroxyl, epoxide, and carboxylic groups. This suggests the presence of nonoxidized graphite in the preparation of graphene oxide. Additionally, sample heterogeneity and variations in the oxidation process can cause peak broadening and overlapping. Measurement conditions and potential residual contaminants on the GO surface may also contribute to the observed spectral characteristics.

The XPS and EDX-SEM data provided in Table 3 highlight the significant changes in the chemical composition of GO and rGO. The higher carbon content and reduced oxygen content in rGO indicate the successful reduction process. The increase in the C/O ratio from 1.95 in GO to 7.31 in rGO (XPS data) further supports this observation, reflecting the restoration of the graphitic structure in rGO.^70,74^ These changes are crucial for understanding the improved efficiency of rGO in isolating nonalbumin proteins, as the reduced oxygen content and restored graphitic structure enhance its interaction capabilities.

### Thermal Analysis and Functional Group Dynamics of Graphene Oxide and Reduced Graphene Oxide

TGA is utilized to elucidate the reduction phenomena by identifying the presence of oxygen functional groups in GO and rGO through the thermal degradation of these materials.^60,75^Table 4 illustrates the weight loss of the samples, with specific steps chosen based on a comprehensive comparison with the derivative weight change (DTG thermogram) presented in Figure S2 [Supporting Information].

**Table 4:** Staged Mass Loss in GO and rGO via Thermogravimetric Analysis

**samples**	**first step, %**	**second step, %**	**final step, %**
GO	17.97	35.67	46.36
rGO	3.92	7.32	88.76

**Figure 6:**
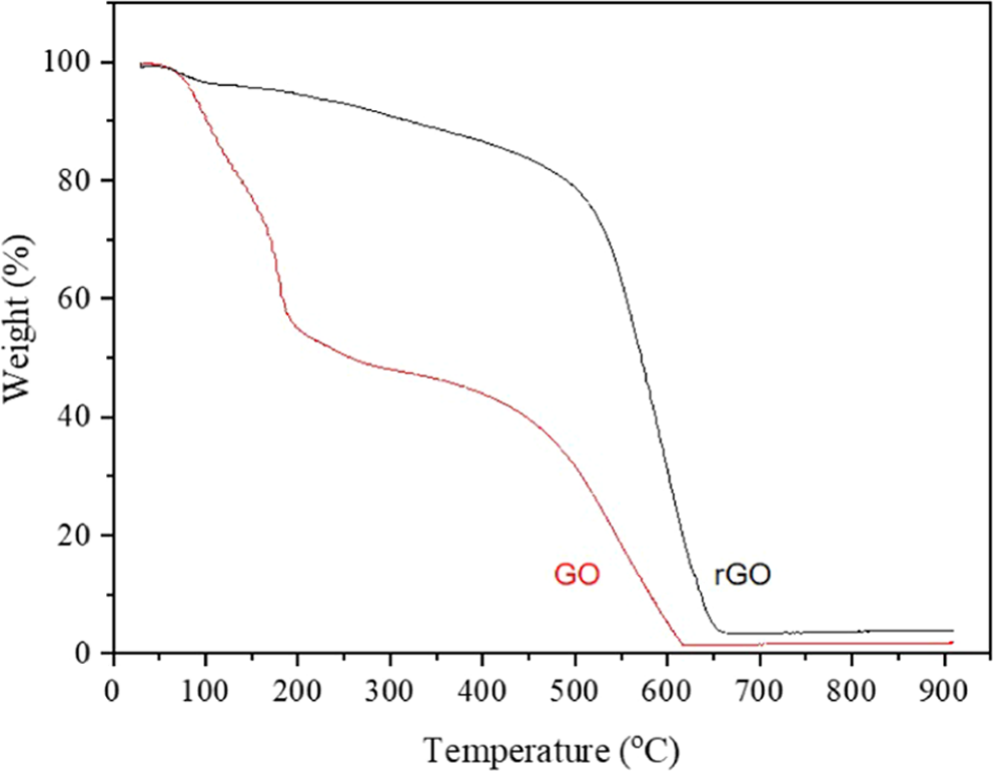
TGA Thermograms of GO and rGO. The graph shows the thermal degradation profiles of GO (red line) and rGO (black line) in an oxygen atmosphere. It illustrates distinct mass loss stages for each material at various temperature ranges, highlighting differences in thermal stability and decomposition behavior between GO and rGO.

The initial step, associated with the loss of adsorbed water, ranges from room temperature (∼30 °C) to 120 °C (Figure 6). rGO exhibits minimal excess water loss compared to GO, which shows significant moisture loss within its structure. The subsequent step, from 120 °C to around 350 °C, corresponds to the removal of oxygen-containing functional groups. The decomposition of each oxygen functionality in GO is observed through a gradual weight loss. The complete thermal decomposition of GO and rGO occurs at approximately 620 and 670 °C, respectively, indicating distinct thermal decomposition patterns. Upon heating, GO exhibits small and two major peaks in the derivative weight near 96, 178, and 553 °C, suggesting two principal mass transitions (Figure S2). In contrast, rGO displays a minor peak near 68 °C (attributed to the removal of water molecules) and a significant peak near 593 °C, related to the oxidative pyrolysis of the carbon framework.^53,58,76^ These findings confirm the effective removal of oxygen functional groups through hydrazine reduction.

The XRD, Raman, TEM, FTIR, XPS, SEM-EDX, and TGA analyses provide a comprehensive understanding of the properties of as-prepared GO and rGO, including their graphitic and crystal structures, chemical composition, impurities, and, notably, functional groups. As Simsikova (2017) and Palmieri (2019) discuss, the rich presence of oxygen functional groups on the GO surface offers numerous sites for attaching molecules such as proteins and enzymes.^36,37^ While GO attracts a variety of proteins, the preference of each protein for GO surface-bound or unbound states is influenced by factors such as pH and the ionic strength of the buffer, affecting the charge status of protein surface functional groups. These insights lay the groundwork for developing a facile albumin isolation method using graphene derivatives. Moreover, the potential role of oxygen-containing functionalities on graphene-based materials in eliminating unwanted serum proteins, such as globulins, is further investigated.

### Development of a Graphene Derivative-Based Albumin Isolation Method

**Figure 7:**
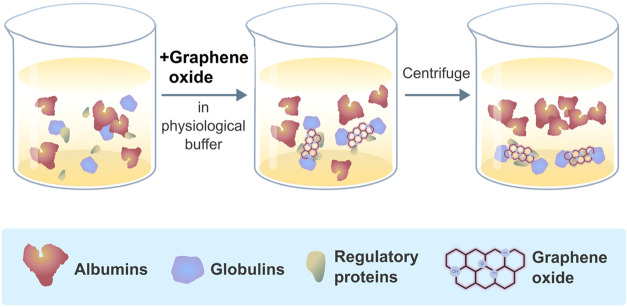
Schematic representation of the albumin isolation process using GO. The illustration demonstrates the sequential steps where GO selectively binds globulins and regulatory proteins in a physiological buffer, allowing for the isolation of purified albumin postcentrifugation.

Albumin, the most abundant protein in the bloodstream among various serum proteins (Table S2, in Supporting Information), is the focus of our newly designed isolation method. This method involves removing serum proteins, such as globulins, by their binding to particles in an isolation reagent, subsequently collecting purified albumin from the supernatant fraction. The cornerstone of our developed method is the utilization of graphene derivatives, leveraging physical adsorption mechanisms, including electrostatic, π–π stacking, and hydrophobic interactions, to establish a green, simple, and effective albumin isolation technique. Furthermore, the albumin isolation method is distinguished by its straightforward application, notably eliminating the need for an elution step and employing environmentally benign reagents, as illustrated in Figure 7.

### Graphene Derivatives in Protein Isolation: Interaction Dynamics and Charge Influences

**Figure 8:**
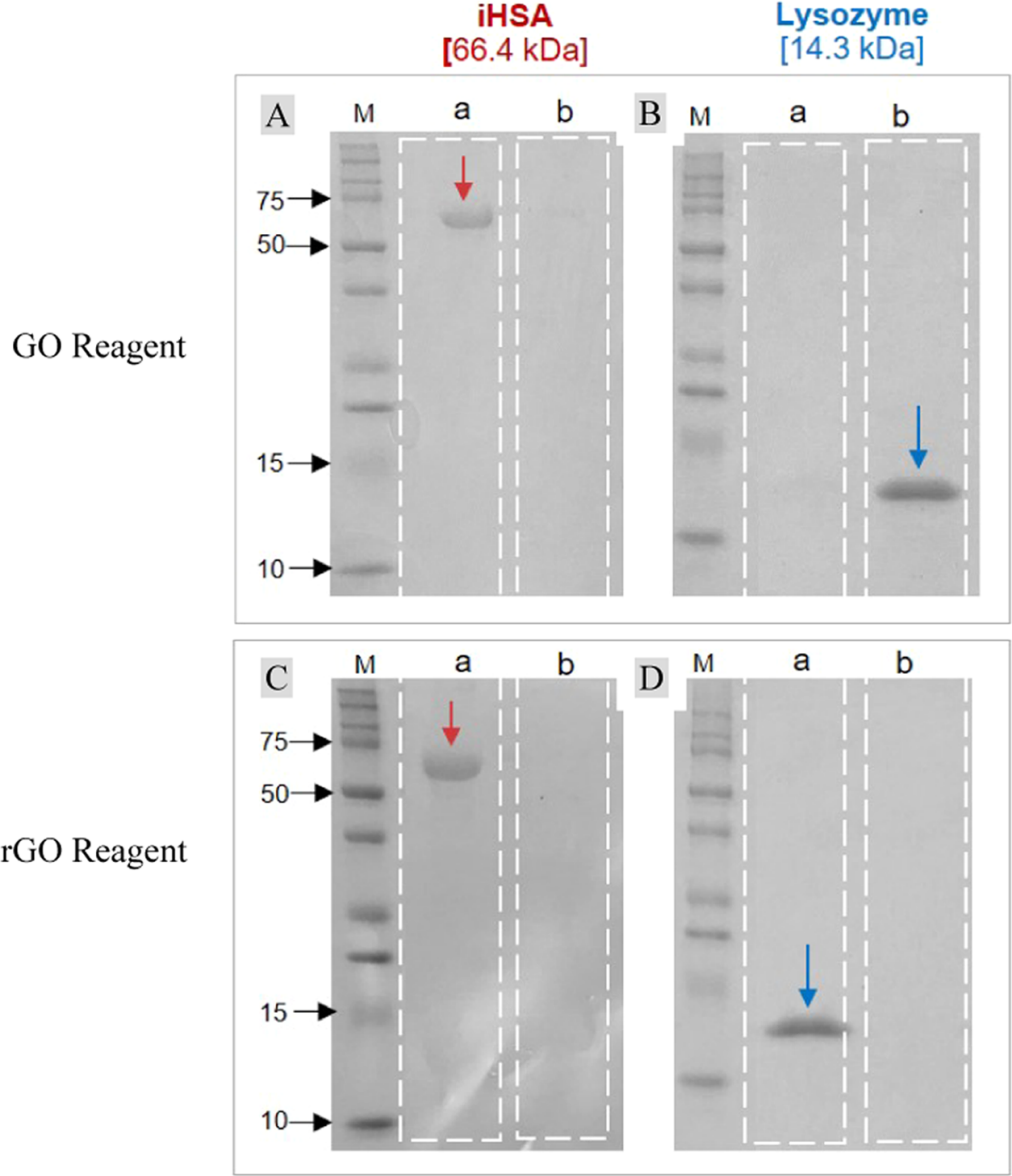
SDS-PAGE analysis for isolation of proteins using GO and rGO reagents. This figure illustrates the separation of a negatively charged protein, iHSA (66.4 kDa), and a positively charged protein, lysozyme (14.3 kDa), through SDS-PAGE. Each lane was loaded with 2 μg of total protein. The assay was performed at pH 7.0, where lysozyme is positively charged, and albumin is negatively charged in the context of the binding assay. However, during SDS-PAGE, both proteins are negatively charged due to SDS binding.

As-prepared materials were dissolved and sonicated for isolation reagent preparation. Preliminary tests reveal that lysozyme (14.3 kDa, pI ≈ 11), which is positively charged under neural condition (pH 7.0), prefers binding to GO rather than rGO, as observed in Figure 8B, lane b. Conversely, isolated-state human serum albumin (iHSA, 66.4 kDa, pI ≈ 4.7), which is negatively charged under neutral condition, was found exclusively in the supernatant fraction (Figure 8A,C, lane a) of both GO and rGO reagents, indicating that iHSA does not bind to these graphene-based materials. However, during SDS-PAGE, both lysozyme and iHSA bind SDS and become negatively charged. DLS (Dynamic Light Scattering) studies corroborate this observation, as shown in Table 5, providing additional insights into the physical interactions between graphene derivatives and proteins. Binding proteins to the surface of particles can alter the surface charge and particle size. Specifically, the change in surface charge density (i.e., ζ potential) of negatively charged particles indicates the adsorption of proteins on the particle surface.

**Table 5:** Surface Charge and Particle Size Alterations in GO and rGO upon Protein Binding: A Dynamic Light Scattering Analysis

**GO-protein binding**	ζ **potential [mV]**	**size distribution [nm]**
GO reagent [bare GO]	–45.0 ± 1.2	1761 ± 375
GO reagent + iHSA	–38.5 ± 1.0	1953 ± 465
GO reagent + lysozyme	–6.6 ± 0.0	3320 ± 686
rGO reagent [bare rGO]	–39 ± 2.0	2860 ± 476
rGO reagent + iHSA	–25.1 ± 8.0	2849 ± 827
rGO reagent + lysozyme	–29.6 ± 0.1	2628 ± 874

The panels show results from using GO reagent isolation (A and B) and rGO reagent isolation (C and D), with lanes labeled (M) for protein markers, (a) indicating supernatant fractions, and (b) representing sediment fractions.

Table 5 presents the relevant findings: the ζ potential of GO decreased to −6.6 ± 0.0 mV, and the hydrodynamic size increased to 3320 ± 686 nm upon lysozyme binding, demonstrating a preference for positively charged proteins on GO. Conversely, the ζ potential and size distribution of GO bound iHSA exhibit slight changes to −38.5 ± 1.0 mV and 1953 ± 465 nm, respectively, compared to bare GO. Furthermore, rGO, distinguished from GO by its reduced content of oxygen-containing functional groups, shows minor changes in surface charge density for positively and negatively charged proteins. Specifically, the less negative zeta potential value for rGO-iHSA compared to pristine rGO can be explained by the adsorption of iHSA onto the rGO surface. When iHSA, which is negatively charged, binds to the rGO, it partially neutralizes the surface charge of rGO. This binding results in a reduction of the overall negative charge on the surface, leading to a less negative zeta potential value. The interaction between the negatively charged functional groups of iHSA and the surface of rGO effectively decreases the surface charge density, hence the less negative zeta potential observed.

Three types of noncovalent interactions, which are van der Waals, electrostatic, and hydrophobic, significantly contribute to protein adsorption on particles.^77^ Baweja’s study suggests that electrostatic interaction predominantly facilitates peptide adsorption on GO, while both electrostatic and van der Waals interactions influence the rGO-peptide system.^78^ These findings align with our results, indicating that both attractive and repulsive electrostatic interactions, mediated by the oxygen-containing functionalities of GO and charged proteins, drive these phenomena.^36,79^ Despite the potential role of hydrophobic interactions in protein adsorption, in comparison to rGO, the deconvoluted C 1s peak of the as-prepared GO at binding energy of 291.3 eV, assigned to π–π* region, show the relatively low intensity (0.01 atom % area) compared to rGO (2.63 atom % area) shown in Table S1, indicating the removal of some of the sp^2^-hybridized carbon structures during the oxidation process, which is a signature of a considerable high degree oxidation resulting the predominantly electrostatic interaction between GO and protein.^78,80^

### Comparison of GO and rGO for Protein Purification

**Figure 9:**
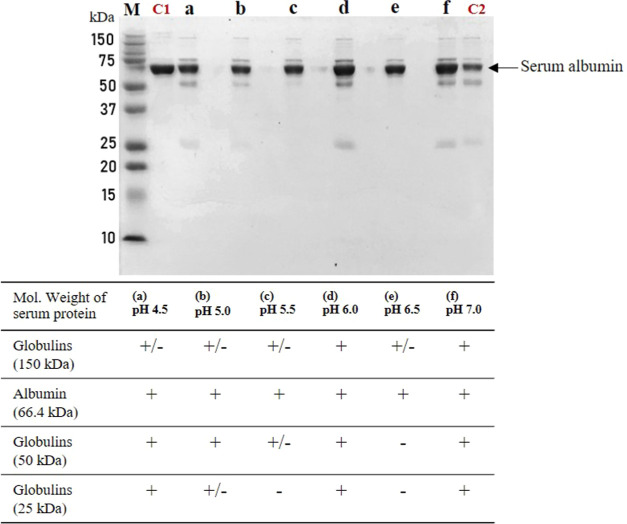
SDS-PAGE analysis of protein isolation across pH conditions. This figure demonstrates the effect of varying pH levels (4.5, 5.0, 5.5, 6.0, 6.5, and 7.0) on the separation of proteins using the GO isolation method. Each lane was loaded with 2 μg of total protein. The lanes marked (a–f) correspond to supernatant fractions obtained with GO reagents at the specified pH values. Lane (M) serves as the protein marker, (C1) is a positive control using iHSA (2 μg), and (C2) features a commercial albumin extraction reagent for comparison. The presence of serum albumin is highlighted across different pH conditions.

**Figure 10:**
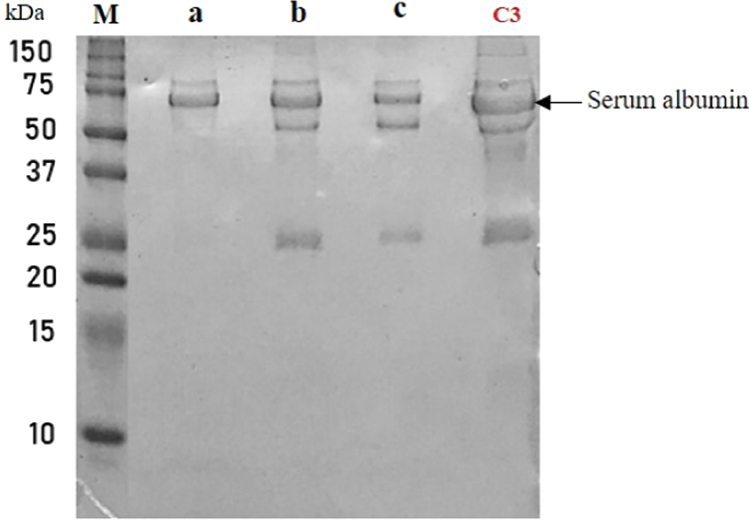
SDS-PAGE Analysis of Albumin Purification Efficacy. This figure compares albumin purification from serum using different methods: (a) GO-based technique, (b) rGO-based technique, (c) commercial reagent kit. Lane M denotes the protein marker, and lane C3 is the control serum sample.

Both GO and rGO were studied in this work to determine their effectiveness as purification materials for albumin isolation. Initially, the suitability of either material for this purpose was unknown. After the preliminary tests (Figure 8 and Table 5), it became evident that GO exhibited superior performance in binding nonalbumin protein compared to rGO. Consequently, GO was selected for further reagent optimization experiments (Figures 9, S3, and S4). Finally, both GO and rGO reagents were tested with biological samples (Figure 10 and Table 6) to evaluate and compare their isolation efficiency. The results demonstrated the absence of globulin bands, the second most abundant serum proteins (Table S2), in the purified sample obtained using the GO method. This indicates the superior performance of GO in achieving high-purity isolation of nonalbumin proteins.

**Table 6:** Isolation Efficacy of Serum Proteins by GO, rGO Methods, and Commercial Kits

	**isolation method**		
**mol. weight of serum protein**	**(a) GO**	**(b) rGO**	**(c) commercial kit**
globulins (150 kDa)	–	±	–
albumin (66.4 kDa)	+	+	+
globulins (50 kDa)	–	+	+
globulins (25 kDa)	–	+	+

The enhanced performance of GO can be attributed to its higher content of oxygen-containing functional groups, which facilitate stronger interactions with nonalbumin proteins, leading to more efficient isolation. Although rGO also possesses residual oxygen-containing functional groups, their number and reactivity are reduced compared to GO, resulting in less efficient binding and isolation. Based on these findings, we conclude that GO is the more suitable material for the extraction of nonalbumin proteins due to its higher efficiency in achieving high-purity isolation. This conclusion is justified by the comprehensive characterization and comparative analysis of the performance of both GO and rGO in our study.

### Influence of pH on Protein Adsorption and Isolation Efficiency Using Graphene Oxide

Altering the charge of particles and proteins can be easily achieved through pH changes, as previously outlined.^81^ Although GO offers numerous reaction sites for various molecules, proteins display specific preferences for binding or not binding to the particle surface, influenced by the pH conditions and the ionic strength of the buffer solution.^82^Figure 9 illustrates the outcomes of adjusting pH values in the isolation reagents. The serum samples purified by the GO reagent were analyzed using 1D SDS-PAGE to identify the proteins remaining in the GO-purified samples. Notably, lanes e (pH 6.5) and c (pH 5.5) exhibit the highest purity of serum albumin. The disappearance of globulin bands around 25, 50 kDa (in reduced form) and 150 kDa (in nonreduced form) suggests the effective removal of these contaminants by the GO reagent at pH 6.5 and 5.5, showcasing the GO method’s efficacy. Lanes C1 and C2 further emphasize this point, with C1 representing samples treated with the GO method and C2 showing those treated with a commercial albumin extraction reagent. The contrast between these lanes demonstrates the superior ability of the GO method to effectively remove globulins, a testament to its advantageous performance over conventional techniques. The isolation reagent at pH 6.5 utilizes PBS buffer, while sodium citrate buffer is employed for preparing the isolation reagent at pH values lower than 6.0. In solution, GO inherently exhibits acidity through its interaction with water, leading to proton generation, bond cleavage, and formation.^83^ Moreover, globulins, the second most abundant serum protein (Table S2, in Supporting Information), generally carry a positive charge at mildly acidic pH conditions^84^ due to their isoelectric points (pI). Thus, the pH value of the isolation reagent plays a crucial role in directly influencing the protein adsorption capability on the particle surface.

### Optimization and Efficacy of the GO-Based Albumin Isolation Method

From the presented results, it is evident that GO exhibits a preference for binding to positively charged proteins over rGO, attributed to the ability of globulins to assume a positive charge through optimal mild acidic pH conditions and ionic strength of the buffer. This finding underscores that globulins can be effectively eliminated under the right pH conditions through electrostatic interactions with GO, akin to the previously studied lysozyme system. Concerning ionic strength, an increase tends to reduce net charge repulsion, leading to enhanced protein aggregation.^85^ Typically, phosphate buffer saline (0.01 M PBS) is utilized as a low ionic strength buffer to mimic physiological conditions, routinely employed in various studies.^82^ Hence, GO in PBS (at pH ∼ 6.5) has been identified as suitable and selected for use in the albumin isolation method. Additionally, the ratio of sample-to-reagent and incubation time were optimized to achieve the highest albumin purity using the GO reagent. The outcomes from these optimization studies, as depicted in Figures S3 and S4, suggest that increasing the reagent volume enhances albumin purity up to a peak ratio of approximately 1:50 for undiluted samples or 1:5 for diluted samples within a minimum incubation time of 15 min [Figures S3 and S4].

The efficiency of the developed albumin isolation method employing graphene derivatives was evaluated in biological samples and benchmarked against commercially available reagents. Table 6 compiles the data from 1D SDS-PAGE analyses used to separate and identify proteins, highlighting the absence of the globulin band in samples treated with the GO method [Figure 10, lane a]. This observation unequivocally demonstrates the removal of globulins from the biological samples, asserting the superiority of the GO method over conventional isolation techniques [Figure 10, lanes b and c]. The commercial reagent was applied following its kit’s recommended protocol, whereas the rGO method adhered to a protocol similar to the GO method.

### Mass Spectrometry Analysis of Albumin Purity and Protein Composition in GO-Isolated Samples

Mass spectrometry (MS) analysis was conducted as described in the experimental section to precisely determine the purified albumin fraction’s protein composition. The results obtained from LC-ESI-MS/MS coupled with MaxQuant analysis, are presented. Table 7 lists the most abundant serum proteins identified in the purified supernatant fraction using the GO method, analyzed by MS/MS. This analysis confirms that the purity of the purified serum albumin from the GO method exceeds 95%.

**Table 7:** Comprehensive Analysis of Serum Protein Composition Post-GO Method Isolation as Revealed by Mass Spectrometry^a^

**protein IDs**	**protein names**	**mol. weight (kDa)**	**iBAQ%**
P02768	serum albumin	69.366	95.32 (±1.01)
P02763	α-1-acid glycoprotein 1	23.539	0.96 (±0.34)
A0A286YEY1;P01876; A0A0G2JMB2;A0A286YEY5; P01877	Ig α-1 chain C region & Ig α-2 chain C region	42.848	0.59 (±0.38)
A0A0A0MS08;P01857; A0A0A0MS07	Ig γ-1 chain C region	43.911	0.47 (±0.11)
P01834;A0A5H1ZRQ3	Ig κ chain C region	11.765	0.39 (±0.31)
P81605	dermcidin	11.284	0.23 (±0.26)
P19652	α-1-acid glycoprotein 2	23.602	0.23 (±0.17)
Q96TA2	ATP-dependent zinc metalloprotease YME1L1	86.454	0.27 (±0.09)
P02787;H7C5E8;C9JVG0	serotransferrin	77.063	0.23 (±0.14)
P01023;H0YFH1;F8W7L3	α-2-macroglobulin	163.29	0.14 (±0.13)
P02647;F8W696	apolipoprotein A-I	30.777	0.13 (±0.12)
P0CF74;A0A5H1ZRQ7; A0M8Q6	Ig λ-6 chain C region; Ig λ-7 chain C region	11.265	0.19 (±0.21)
H3BP51;Q8NEP3	dynein assembly factor 1, axonemal	27.695	0.12 (±0.06)
P01009;A0A024R6I7; A0A0G2JRN3;G3 V2B9; G3 V544	α-1-antitrypsin	46.736	0.08 (±0.11)
Q06609;E9PNT5;E9PJ30	DNA repair protein RAD51 homologue 1	36.966	0.04 (±0.02)

a The proteins are ordered by relative abundance calculated by iBAQ values as analyzed by MaxQuant 2.2.0.0.

**Figure 11:**
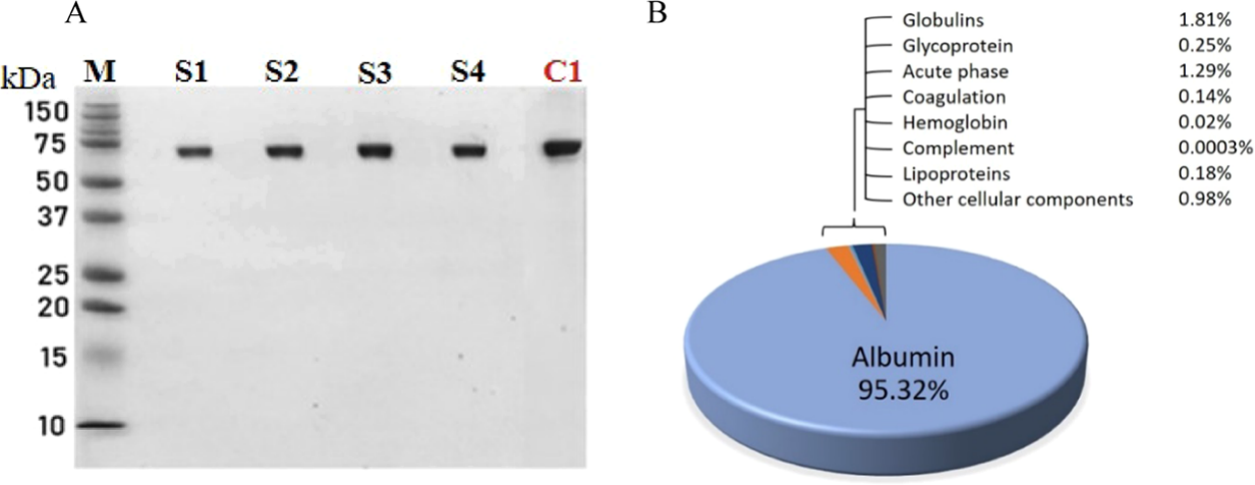
SDS-PAGE and proteomic analysis of serum samples using the GO method. (A) SDS-PAGE of supernatant fractions obtained through the GO method, with lanes labeled (M) for the protein marker and (S1–S4) representing albumin extracted from various serum samples using the GO reagent. Lane (C1) serves as the isolated-state human serum albumin (iHSA) positive control at 2 μg. (B) Pie chart detailing the protein composition of the purified supernatant fractions from four serum samples, as determined by mass spectrometry, categorized by protein function. The chart highlights the significant dominance of albumin in the purified fractions.

Despite the protein profile for the GO method (Figure 11A) displaying only a single albumin band, suggesting near 100% purity, the presence of other serum proteins in the purified sample is evident through MS/MS analysis. While 1D SDS-PAGE is commonly employed for identifying protein purity and monitoring purification processes,^86^ it is recommended to complement it with MS analysis for a more accurate determination.^43^

The pie chart in Figure 11B illustrates the statistical distribution of proteins, organized by function, in the purified serum samples obtained through the GO method. From this chart and Table 7, several conclusions can be drawn: (i) the GO method achieved 95% purity of purified serum albumin using mild reagent conditions and a simple one-step process; (ii) a residual presence of globulins, about 1.8%, was noted, with over 1.5% attributed to α-1 globulin. Globulins are typically classified into four groups, including α-1, α-2, β, and γ, by electrophoresis,^87^ with normal adult levels ranking from highest to lowest as γ > β > α-2 > α-1. This suggests that the minimal presence of α-1 globulin, rather than the more abundant γ globulin (with a pI of 7.2), is significant.^88^ The charge properties of these proteins, where negatively charged proteins are prevalent at pH values above their pI, indicate that α-1 globulin (pI = 5.1) is negatively charged under the pH conditions of the GO method. In contrast, γ globulin, being positively charged, is more inclined to bind to the GO surface through attractive interactions than α-1 globulin.

The GO-based method achieves high purification efficiency without the need for hazardous chemicals, offering a green, cost-effective, and simple alternative suitable for clinical applications. The results demonstrate that this method can isolate albumin with over 95% purity, as confirmed by SDS-PAGE and mass spectrometry (Figures 11A and Table 7). The comparison of the initial serum sample (Figure 10, lane C3) with the purified albumin sample highlights the effectiveness of our method in reducing contaminant proteins.

The same lot of as-prepared GO and rGO was utilized throughout our study, which extended over a period of more than one year, thereby demonstrating the materials’ stability. Reusability was not assessed in this study; however, while it is theoretically feasible to reuse GO and rGO after removing unwanted serum proteins and replenishing with new buffer, the process is labor-intensive and may not be cost-effective if fresh reagents are relatively inexpensive. The biocompatibility and toxicity of graphene-based materials are known to depend on their functionalization, which can mitigate toxic effects. In the context of this study, GO and the isolated proteins are not intended for direct human use. Nonetheless, comprehensive exploration of these issues is warranted in future projects to ensure safety and efficacy for potential clinical applications.

A potential limitation of our methodology is the presence of low molecular weight compounds in the supernatant containing purified albumin. Although our GO-based method effectively isolates high-purity albumin, as demonstrated by the SDS-PAGE analysis (Figure 11A) and confirmed by mass spectrometry (Table 7), there is a possibility that low molecular weight compounds from the plasma may remain in the supernatant. The initial serum sample, shown in Figure 10 (lane C3), displays several contaminant proteins, highlighting the complexity of the plasma composition. To provide a more robust evaluation of our GO-based method, it is essential to compare it directly with established albumin purification techniques, such as Blue Sepharose chromatography, which is known for achieving high-purity albumin through affinity interactions. This direct comparison will allow us to more accurately assess the efficacy of our method relative to existing standards, and will be a key focus of our future studies.

In summary, while our GO-based method successfully achieves over 95% purity for isolated albumin, it is crucial to address the presence of low molecular weight compounds in the supernatant for clinical applications. Moving forward, our research will focus on integrating additional purification techniques to further improve the purity of HSA, ensuring it meets the highest standards for clinical use. We aim to simplify the process while maintaining or enhancing the method’s effectiveness, making it more suitable for widespread application.

## Conclusions

This investigation successfully establishes a green, straightforward, and effective albumin isolation method utilizing GO, setting a benchmark for simplicity and environmental friendliness in protein purification processes. Our method capitalizes on GO’s inherent physicochemical properties to achieve selective albumin extraction from serum with high purity levels, as verified by SDS-PAGE and proteomic analysis. The optimized conditions of pH and incubation time further enhance the method’s efficiency, providing a scalable solution to meet clinical and research demands. This study underscores the potential of graphene derivatives in biomedical applications and contributes to the broader quest for sustainable and efficient biochemical processing techniques. Future research could explore the scalability of this approach and its applicability across various biological matrices, potentially revolutionizing the way albumin and other proteins are purified for medical and research purposes.

## Supporting Information Available

Figure S1. Scanning electron microscopy (SEM) and energy dispersive X-ray spectroscopy (EDX) analyses of GO and rGO. Figure S2. Thermogravimetric analysis and differential thermogravimetry (TGA-DTG) profiles for GO and rGO. Figure S3. SDS-PAGE analysis for optimizing the sample-to-reagent volume ratio in albumin isolation via GO method. Figure S4. SDS-PAGE analysis of albumin isolation efficiency across different incubation times using the GO method. Table S1. C 1s and O 1s Deconvolution of GO and rGO. Table S2. Serum and plasma protein composition with relative abundance.^89^ (PDF)

## Abbreviations

GO: graphene oxide
rGO: reduced graphene oxide
HSA: human serum albumin
BSA: bovine serum albumin
SDS-PAGE: sodium dodecyl sulfate polyacrylamide gel electrophoresis
TEM: transmission electron microscopy
SEM: scanning electron microscopy
XRD: X-ray diffraction
FTIR: Fourier transform infrared spectroscopy
XPS: X-ray photoelectron spectroscopy
TGA: thermogravimetric analysis
DLS: dynamic light scattering
PBS: phosphate-buffered saline
LC-ESI-MS/MS: liquid chromatography electrospray ionization tandem mass spectrometry
