# Supplementary material for: Advancing Albumin Isolation from Human Serum with Graphene Oxide and Derivatives: A Novel Approach for Clinical Applications
Source: ACS Omega. 2024 Sep 20;9(39):40592–607. doi: 10.1021/acsomega.4c04276 (PMC11447712; doi:10.1021/acsomega.4c04276)
Supplement: Supplementary file 1 — ao4c04276_si_001.pdf [file ao4c04276_si_001.pdf]

Supporting information

Advancing Albumin Isolation from Human Serum with Graphene Oxide and Derivatives: A Novel Approach for Clinical Applications

Chayachon Apiwat [1, 3], Jack W Houghton [5], Ren Ren [5, 6], Edward Tate [5], Joshua B Edel [5], Narong Chanlek [4], Patraporn Luksirikul [1,2], Deanpen Japrung [3*]*

[1] Department of Chemistry, Faculty of Science, Kasetsart University, Bangkok, Thailand

[2] Center for Advanced Studies in Nanotechnology for Chemical, Food and Agricultural Industries, KU Institute for Advanced Studies, Kasetsart University, Bangkok, 10900, Thailand

[3] National Nanotechnology Center (NANOTEC), National Science and Technology Development Agency (NSTDA), Thailand Science Park, Pathumthani, Thailand

[4] Synchrotron Light Research Institute (Public Organization), 111 University Avenue, Muang, Nakhon Ratchasrima 30000, Thailand

[5] Department of Chemistry, Molecular Sciences Research Hub, Imperial College London, London, UK.

[6] Department of Metabolism, Digestion and Reproduction, Imperial College London, London, UK.

* Corresponding authors: Patraporn Luksirikul (email address: fscipplu@ku.ac.th) and Deanpen Japrung (email address: deanpen@nanotec.or.th)

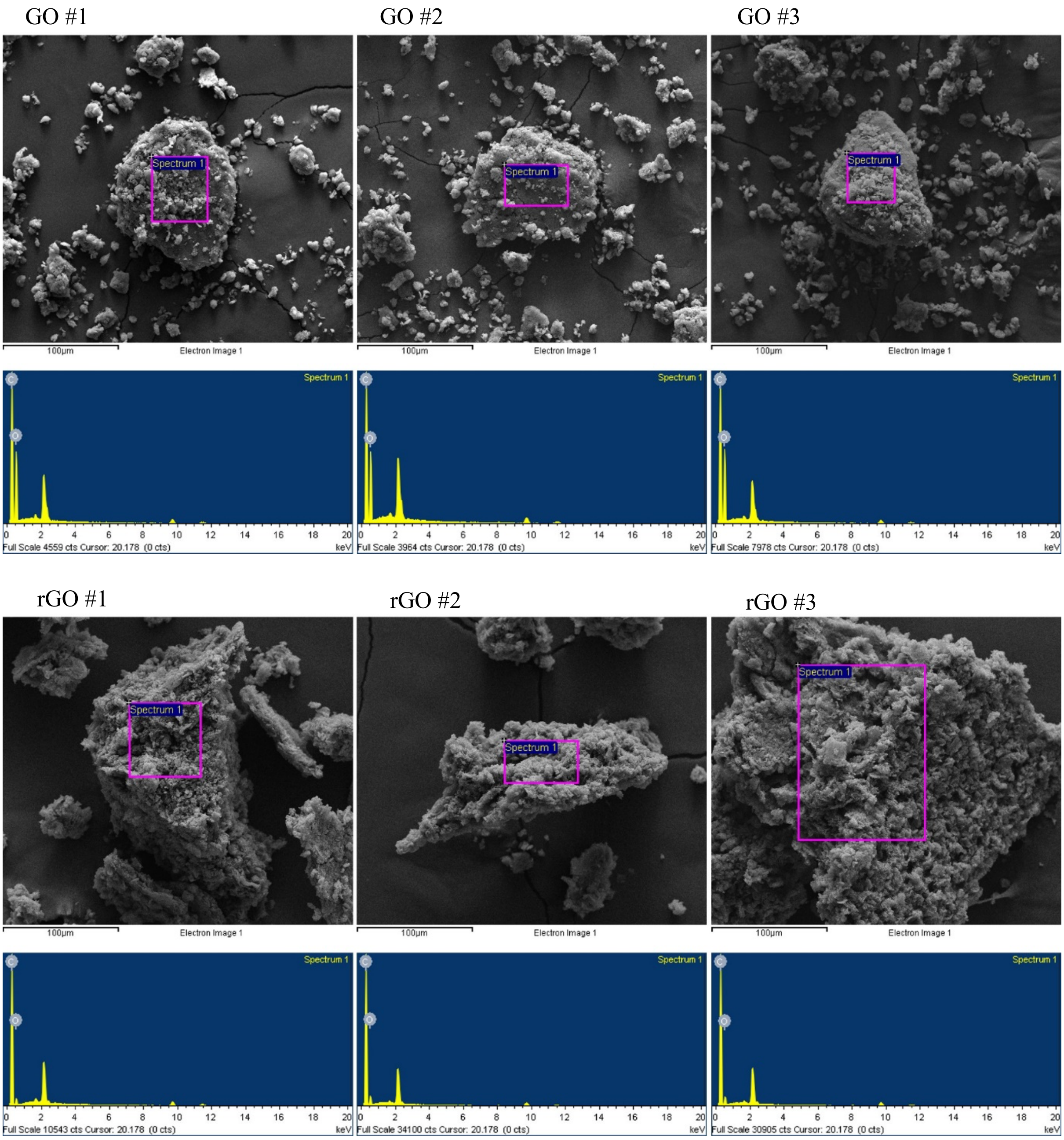

Figure S1. Scanning Electron Microscopy (SEM) and Energy-Dispersive X-ray Spectroscopy (EDX) Analyses of Graphene Oxide (GO) and Reduced Graphene Oxide (rGO). SEM images display the microstructural details of GO and rGO, with selected areas highlighted for EDX analysis. Corresponding EDX spectra, obtained from the highlighted regions in each SEM image, illustrate the elemental composition. Images and spectra are shown in triplicate to ensure replicability of the observed features.

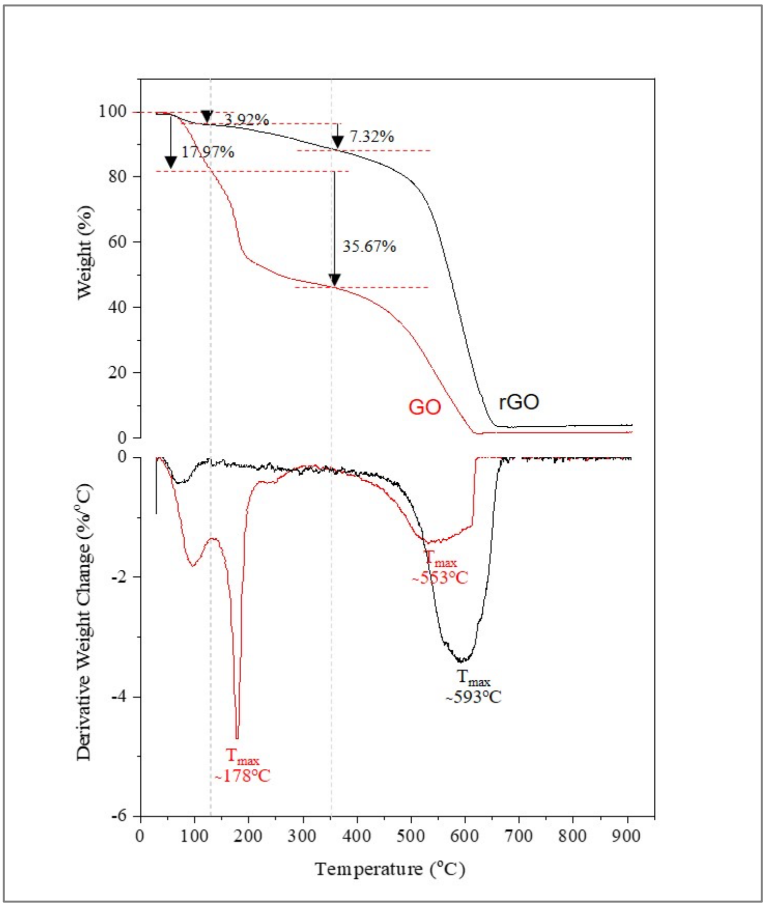

Figure S2. Thermogravimetric Analysis and Differential Thermogravimetry (TGA-DTG) Profiles for GO and rGO. The figure displays the thermal degradation behavior and associated weight loss percentages of graphene oxide (GO, indicated by the red line) and reduced graphene oxide (rGO, shown in black) across various temperature stages. Key temperature points leading to maximal rate changes in weight (T_{max}) are highlighted for both materials, showcasing their distinct thermal stability and degradation pathways.

Table S1. C 1s and O 1s Deconvolution of GO and rGO.

Sample	C 1s Deconvolution (at. %area)						O 1s Deconvolution (at. %area)				C-OH/ C-O-C
	sp^2 C	sp^3 C	C-O	C=O	O-C=O	π-π*	C-O	C=O	C-OH	C-O-C	
GO	284.4 (eV)	285.4 (eV)	286.5 (eV)	288.1 (eV)	289.8 (eV)	291.3 (eV)	531.5 (eV)	532.5 (eV)	533.7 (eV)	534.7 (eV)	
	14.36	16.02	33.45	30.74	5.43	0.01	7.54	36.31	38.93	17.21	2.59
rGO	284.4 (eV)	285.4 (eV)	286.5 (eV)	288.1 (eV)	289.8 (eV)	291.3 (eV)	531.3 (eV)	532.4 (eV)	533.6 (eV)	-	-
	65.32	12.70	11.50	7.59	2.89	2.63	37.38	34.11	28.51	-	

Table S2. Serum and Plasma Protein Composition with Relative Abundance.[89]

Fraction	Type of protein	Abundance
Serum & Plasma	Albumin	55%
Serum &Plasma	Globulins<br>(Alpha-1, Alpha-2, Beta, Gamma)	38%
Plasma	Fibrinogen	7%
Plasma	Clotting factors	< 1%
Serum & Plasma	Regulatory proteins	< 1%

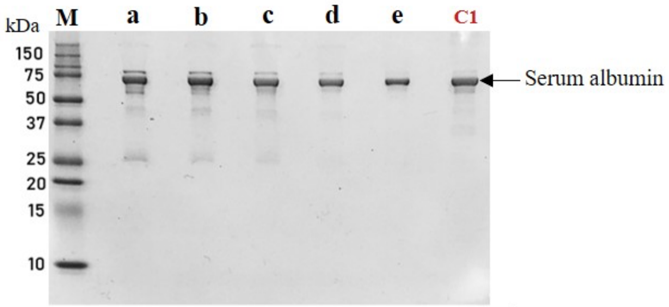

Mol. Weight of serum protein	(a) 1:1	(b) 1:2	(c) 1:3	(d) 1:4	(e) 1:5
Globulins (150 kDa)	-	-	-	-	-
Albumin (66.4 kDa)	+	+	+	+	+
Globulins (50 kDa)	+	+	+	+/-	-
Globulins (25 kDa)	+	+	+	+/-	-

Figure S3. SDS-PAGE Analysis for Optimizing the Sample-to-Reagent Volume Ratio in Albumin Isolation via GO Method. This figure showcases SDS-PAGE results for supernatant fractions obtained by applying the GO method under varying conditions, specifically adjusting the volume ratio of GO reagent (2 mg/mL) to biological sample. The samples were fixed at either 20 µL (for a diluted 1:10 sample) or 2 µL (for an undiluted sample). The lanes are marked as follows: (M) represents the protein marker, (a-e) correspond to incremental GO reagent volumes of 20, 40, 60, 80, and 100 µL respectively applied in the GO method, and (C1) denotes the iHSA positive control at 2 µg, serving as a benchmark for albumin identification.

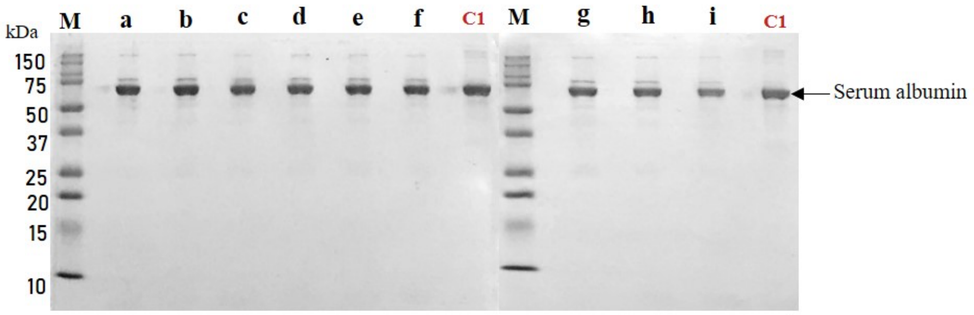

Mol. Weight of serum protein	(a) 15min	(b) 30min	(c) 1 h	(d) 2 h	(e) 5 h	(f) 8 h	(g) 12 h	(h) 16 h	(i) 24 h
Globulins (150 kDa)	+/-	+/-	+/-	+/-	+/-	+/-	+/-	+/-	+/-
Albumin (66.4 kDa)	+	+	+	+	+	+	+	+	+
Globulins (50 kDa)	-	-	-	-	-	-	-	-	-
Globulins (25 kDa)	-	-	-	-	-	-	-	-	-

Figure S4. SDS-PAGE Analysis of Albumin Isolation Efficiency Across Different Incubation Times Using the GO Method. This figure presents SDS-PAGE results for supernatant fractions obtained by applying the GO method under varied incubation times, with a constant GO stock solution concentration (2 mg/mL) and a fixed volume ratio of biological sample (serum diluted 1:10 in buffer solution) at 40 µL to GO reagent at 20 µL. Lanes are denoted as follows: (M) indicates the protein marker, (a-i) represent the series of incubation times [15 minutes, 30 minutes, 1 hour, 2 hours, 5 hours, 8 hours, 12 hours, 16 hours, and 24 hours respectively] explored to assess the optimal duration for albumin isolation, and (C1) is the iHSA positive control at 2 µg, serving as a reference for albumin detection.
